# Supplementary material for: Empirical methods for controlling false positives and estimating confidence in ChIP-Seq peaks
Source: BMC Bioinformatics. 2008 Dec 5;9:523. doi: 10.1186/1471-2105-9-523 (PMC2628906; doi:10.1186/1471-2105-9-523)
Supplement: Additional File 1 — A variety of html documents from the USeq web site detailing the available applications, their best usage, output file type descriptions, command line menus, etc. [file 1471-2105-9-523-S1.zip › USeqUserGuides/JavaDoc.zip!/JavaDoc/cgar/beans/class-use/LabGroupBean.html]

Uses of Class cgar.beans.LabGroupBean


|  |  |  |
| --- | --- | --- |
| **Overview** \| **Package** \| **Class** \| **Use** \| **Tree** \| **Deprecated** \| **Index** \| **Help** | |  |
| PREV   NEXT | **FRAMES**    **NO FRAMES**     **All Classes** |  |


---


## **Uses of Class cgar.beans.LabGroupBean**

No usage of cgar.beans.LabGroupBean

---


|  |  |  |
| --- | --- | --- |
| **Overview** \| **Package** \| **Class** \| **Use** \| **Tree** \| **Deprecated** \| **Index** \| **Help** | |  |
| PREV   NEXT | **FRAMES**    **NO FRAMES**     **All Classes** |  |


---
